# Ultrastructural Damage of *Loligo vulgaris* and *Illex coindetii* statocysts after Low Frequency Sound Exposure

**DOI:** 10.1371/journal.pone.0078825

**Published:** 2013-10-15

**Authors:** Marta Solé, Marc Lenoir, Mercè Durfort, Manel López-Bejar, Antoni Lombarte, Michel André

**Affiliations:** 1 Laboratory of Applied Bioacoustics, Technical University of Catalonia, Barcelona, Spain; 2 INSERM U.1051, Institute of Neurosciences of Montpellier, Montpellier, France; 3 Department of Cellular Biology, Faculty of Biology, University of Barcelona, Barcelona, Spain; 4 Department of Animal Health and Anatomy, Faculty of Veterinary Science, Universitat Autònoma de Barcelona, Campus de la UAB, Bellaterra (Cerdanyola del Vallès), Spain; 5 Renewable Marine Resources Department, Marine Sciences Institute (ICM-CMIMA-CSIC), Barcelona, Spain; Pacific Northwest National Laboratory, United States of America

## Abstract

There is a considerable lack of information concerning marine invertebrate sensitivity to sound exposure. However, recent findings on cuttlefish and octopi showed that exposure to artificial noise had a direct consequence on the functionality and physiology of the statocysts, sensory organs, which are responsible for their equilibrium and movements in the water column. Owing to a lack of available data on deep diving cephalopod species, we conducted a noise exposure comparative experiment on one Mediterranean squid, *Illex coindetii*, and on the European squid *Loligo vulgaris*. Scanning electron microscopy (SEM) revealed similar injuries in the inner structure of the statocysts, as those found in cuttlefish and octopi. In addition to the ultrastructural description of the lesions, we publish here the first images of the *crista*-*cupula* system and inner statocyst cavity of *I. coindetii*.

## Introduction

Although marine mammals [1–6] and fishes [7–10] have originally attracted most of the research attention on the effects of noise on the oceanic ecosystem, invertebrate sensitivity to noise and possible negative effects after sound exposure has also been addressed by several authors [11–19]. A detailed literature review on these effects can be found in recent publications [20,21]. 

**Figure 1:**
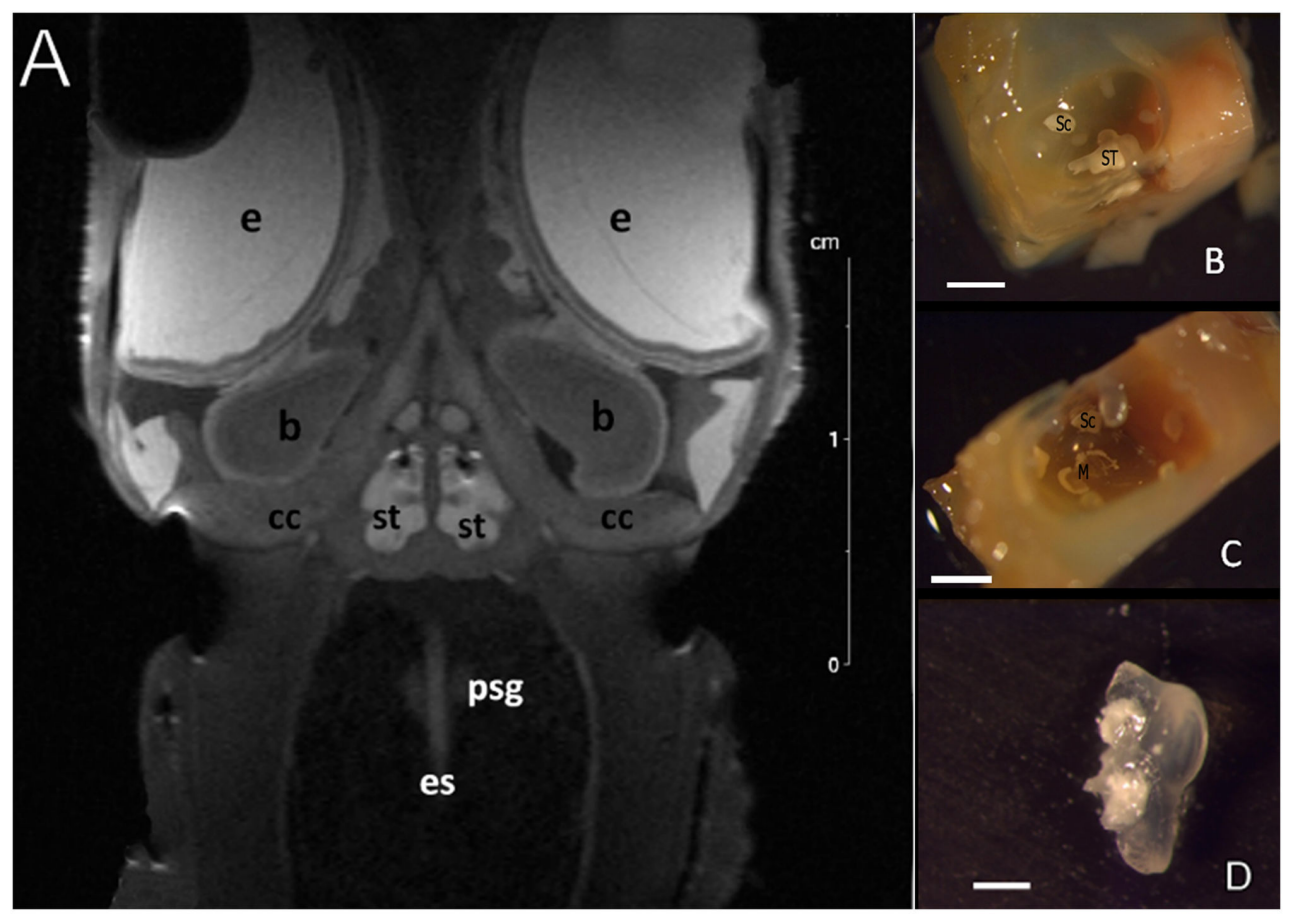
RMI (A) and LM (B-D). **Decapods statocyst location in the cephalic cartilage. A**: Coronal view –anterior section- of squid (*L. vulgaris*) head. **B**, **C**: **Photomicrograps of decapod statocyst structure**. Upper view in the opened *Loligo* *vulgaris* statocyst. **B** shows the statocyt attached to the *macula* *statica* *princeps* and a *statoconia* attached to the superior *macula* *neglecta*. In **C** the statocyst was removed and the *macula* *statica* *princeps* is visible. **D**: *L. vulgaris* statolith. (**B**: Brain, cc: cranial cartilage, e: eye, es: oesophagus, m: mouth, psg: posteror salivary gland, st: statocyst. *ST: Statolith. Sc: statoconia. M: Macula statica princeps*). **Scale bars: A** = 2 cm. **B, C** = 2 mm. **D** =1 mm.

It was suggested [20,21] that cephalopods sensory organs, the statocysts, were presumably the best candidates to injury if exposed to loud sound sources. Indeed, all cephalopods have two statocysts generally located within the cephalic cartilage (Figure 1). The statocyst morphology and its functions have been extensively described elsewhere by different authors [22–28]. The statocysts are specialized balloon-shape bodies filled with endolymph that contain the sensory hair cells. These cells lie on the inside wall of the inner sac and are grouped into two main areas of the sensory epithelium: the *macula*-statolith system and the *crista*-*cupula* system. These systems have clear similarities to the analogous vertebrate vestibular system and present a weight-lending mass and an epithelial layer containing small supporting cells as well as large sensory hair cells [28]. However, unlike ciliated cells of the latter species, the cephalopods’ statocyst sensory cells carry multiple kinocilia. Surrounding the base of the kinocilium are microvilli. Kinocilia and microvilli form elongated bundles. Each bundle represents a single hair cell. Adjacent accessory structures (statolith, statoconia, *cupula*) are responsible for sensory perception. When there is a stimulus, tiny deflations occur in the hair bundles, resulting in cell body depolarization and subsequent transmission of information to the sensory nervous system. Within the central nervous system, the sensory input of the statocysts is used to regulate a wide range of behaviours, including locomotion, posture, control of eye movement and of the pattern of the body coloration, and are suspected to be responsible for the reception of the low frequency sound waves [25,29,30]. The sensory epithelia of the gravity receptor system, in resemblance to the vertebrate auditory apparatus [31] have, in addition to primary hair cells, secondary sensory hair cells, which are unidirectional morphologically and physiologically polarized, first-order afferent neurons, and efferent nerve fibres. The efferent fibres of the statocyst terminate both on hair cells and the axons of afferent neurons [32,33]. 

When exposed to relatively low intensity low frequency sounds, Controlled Exposure Experiments (CEE) revealed lesions which took place in the sensory epithelia of the statocysts’ inner structures of the common Mediterranean cuttlefish (*Sepia officinalis*) and common octopus ( *Octopus vulgaris*) [20,21]. The aim of the present study was to contribute to a better understanding of the effects of noise on marine invertebrate sound reception by comparatively describing the ultrastructure of *Loligo vulgaris* and *Illex coindetii* statocyst sensory epithelium after exposure to the same stimuli. 

Since *L. vulgaris* (Family *Loliginidae*) and *I. coindetii* (Family *Ommastrephidae*) are decapod cephalopods, the ultrastructure of their statocyst is indeed very similar to *S.officinalis* [21]. The ecology of these species is, however, very different. *S. officinalis*, a benthic species, can reach a maximum depth of 200m while *L. vulgaris*, a neritic species, descends down to 550m. *I. coindetii* usually lives at depths of between 100 and 400m, but is commonly found deeper, at 1100m [34]. Would this difference in behavior and physiology, in particular the resistance to high pressures, be reflected in the ultrastructure of these squid sensory cells? And would noise affect them similarly as described in *S. officinalis*? 

## Materials and Methods

### Cephalopod specimens

**Figure 2:**
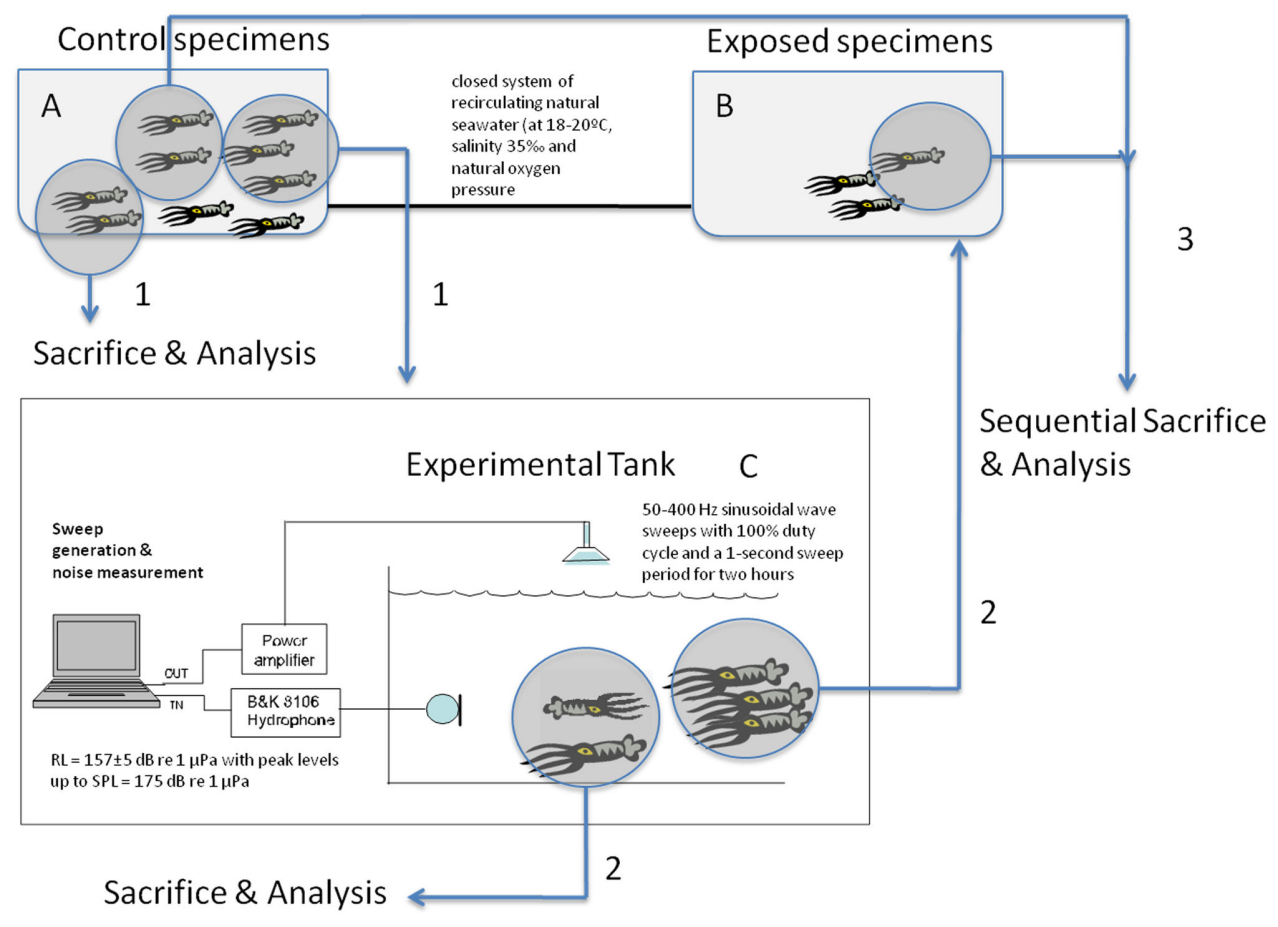
Scheme of the general protocol of the exposure to sound and posterior analyses [21].

Nine individuals from *L. vulgaris* (mantle length 15-25 cm corresponding to 7 adults and 2 sub-adults) and four *I. coindetii* (mantle length 10-13 cm, all adults), were obtained from the Catalan coast (NW Mediterranean Sea) between February of 2008 and August of 2010, and kept in a closed system of recirculating natural seawater (at 18-20°C, salinity 35‰ and natural oxygen pressure) consisting of 2 mechanically filtered fiberglass reinforced plastic tanks with a capacity of 2000L, that were connected to each other (LAB - UPC, Vilanova i la Geltrú) (Figure 2). This included a physicochemical self-filtration system with activated carbon and sand, driven by a circulation pump. 

Individuals were supplied with live crab (*Carcinus maenas*) food ad libitum and were maintained in the tank system until the exposure. Several specimens (see below) were used as controls and were kept in the same conditions as the experimental animals until being exposed to noise [21]. 

### Sound Exposure Protocol

Sequential Controlled Exposure Experiments (CEE) were conducted on adult individuals (n=5) *L. vulgaris* and (n=2) *I. condietii*. An additional set of 2 adult and 2 sub-adult individuals of (n=4) *L. vulgaris* and (n=2) *I. condietii* was used as a control. The same sequential CEEs were conducted as with other cephalopods spp. studied in this project [20,21]. The difference here is that, since the results from the analysis with *S. officinalis* showed lesions immediately after noise exposure, and incremental effects up to 96 hours (longest period of observation), we concentrated the study on animals sacrificed immediately after and 48 hours after exposure, thus reducing the number of specimens used in the experiments**.**

Individuals were maintained in the tank system (tank A) until the exposure. The exposure consisted of a 50-400 Hz sinusoidal wave sweeps with 100% duty cycle and a 1-second sweep period for two hours. The sweep was produced and amplified through an in-air loudspeaker while the level received was measured by a calibrated B&K 8106 hydrophone (RL = 157±5 dB re 1 μPa with peak levels up to SPL = 175 dB re 1 μPa). Some of the animals were used as controls and were kept in the same conditions as the experimental animals until the latter were exposed to noise, in an independent tank (C). The sacrificing process was identical for controls and exposed animals. After the exposure, the individuals that were not immediately sacrificed were placed in tank B (see Figure 2 and sequence of sacrifices below). The independent experimental tank (C) was located in a separate location, acoustically isolated from tanks A and B. Following exposure, the samples (Figure 2) were obtained from the individuals (exposed and controls) at the above intervals. 

As stated in previous publications [20,21], it must be reiterated here that the experiment was not set up to find specific threshold levels, but designed to investigate if these two squid species would present similar acoustic lesions, as found in *S. officinalis* and *O. vulgaris*, when they were exposed to low frequency sounds. Indeed, the non-even distribution of sounds in the experimental tank associated tothe free movement of the exposed animals made impossible to define proper received levels during exposure. In addition, particle motion was not measured, for its importance in the acoustic trauma mechanism could not be determined. Particle motions associated with the acoustic pressures in the experimental setup were most likely higher than the particle motions that would be found accompanying similar acoustic pressures produced in natural sea conditions. Therefore, the measured levels during the experiment cannot immediately be taken as reference values triggering lesions in these species. Future research should map in-tank acoustic pressures, quantify particle motions and reproduce the experiments in open ocean conditions before a definitive conclusion is drawn on the relationship between sound source levels and observed acoustic trauma. 

### Removal of statocysts

In all experiments, isolated head preparations were obtained by decapitation. The experimental protocol strictly followed the necessary precautions to comply with the current ethical and welfare considerations when dealing with cephalopods in scientific experimentation [35]. The statocysts with their surrounding cartilage were extracted and fixed for observation and analysis. For fixation, the statocyst cavity was opened and special care was taken to prevent mechanical damage to the inner tissues. The analysis was performed on tissues obtained from left and right statocysts.

### Imaging Techniques

The same imaging techniques were used as with *S. officinalis* and *O. vulgaris* [20,21]: individuals were processed according to routine SEM procedures. No quantification of the lesions was performed since no reference values, both in terms of acoustic pressure and particle motion, were available (see above sound exposure protocol section): because of the non-evenly distribution of the acoustic pressure into the tank, the specimens were probably exposed to different levels of acoustic pressure (and particle motion) therefore no results are presented here on the corresponding fraction of hair cells that were damaged; on the number of kinocilia that were lost; nor on the number of cells exhibiting swollen endoplasmic reticula. The results section will thus concentrate on a qualitative ultrastructural description of the sensory epithelia.

### Light microscopy (LM)

In addition to the statocysts extraction, a routine necropsy was conducted, to collect samples of different tissues from controls and exposed individuals, which were further fixed in 10% formalin, sectioned, stained with methylene blue, covered with Durcupan and observed using Olympus CX41 light microscope. This analysis was conducted to determine the presence of lesions in mantle surface epithelia, inner muscular fibers of collagen, various organs of the digestive tract, the circulatory, nervous, sensory, respiratory, reproductive and excretory systems and the ink gland complex. 

### Scanning electron microscopy

Eighteen statocysts from 9 *L. vulgaris* and eight statocysts from 4 *I. coindetii* were used for this study. Fixation was performed in glutaraldehyde 2, 5 % for 24-48h at 4°C. Statocysts were dehydrated in graded alcohol solutions and critical-point dried with liquid carbon dioxide in a Leica EmCPD030 unit (Leica Mycrosystems, Austria). The dried statocysts were cut open and flattened out to expose the statocyst structures and then mounted on specimen stubs with double-sided tape. The mounted tissues were gold-palladium coated with a Polaron SC500 sputter coated unit (Quorum Technologies, Ltd.) and viewed with a variable pressure Hitachi S3500N scanning electron microscope (Hitachi High-Technologies Co., Ltd, Japan) at an accelerating voltage of 5kV in the Institute of Marine Sciences of The Spanish Research Council (CSIC) facilities. 

## Results

### Light microscopy (LM)

None of the organs showed any post-mortem artifacts nor any specific lesion except light hemorrhaging in two individuals (not shown here), at mantle level, probably due to impacts against the tank walls during handling operations. Apart from statocysts (see data below), the systematic comparison of the histological preparations between exposed individuals and controls did not reveal the presence of pathology associated with sound exposure in any of the tissues analyzed.

### Structural and ultrastructural investigations of the statocyst sensory epithelium

Regardless of the species, all exposed individuals presented the same lesions in the statocyst sensory epithelia and the same incremental effects versus time 

### *Loligo vulgaris* and *Illex coindetii macula*

**Figure 3:**
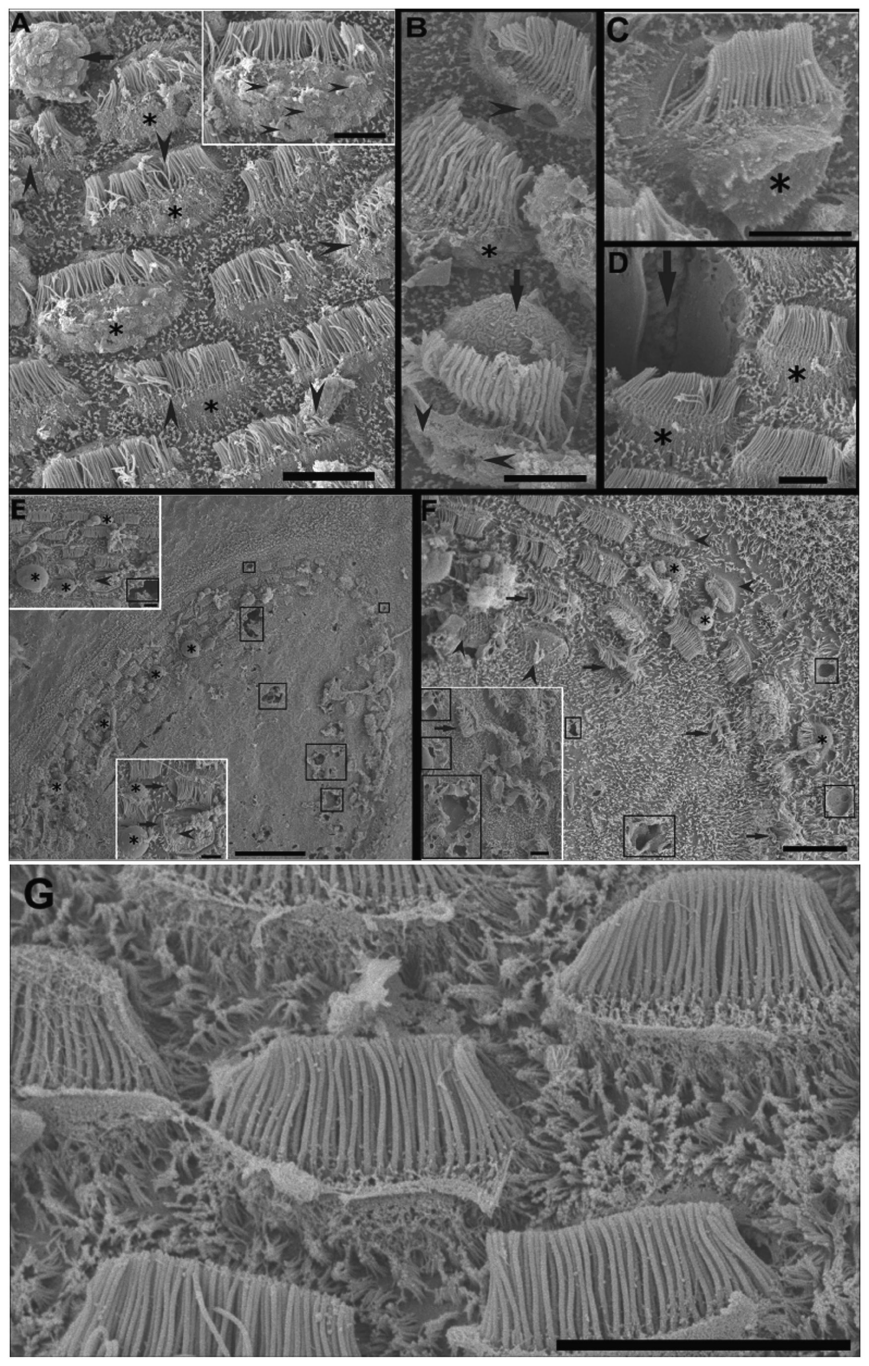
SEM. ***Loligovulgaris* macula statica princeps (msp) sacrificed immediately (A-C). *Illex coindetii* msp sacrificed 48h (E-F) after sound exposure and control animal (G). A**: The surface of the epithelium presents deformation of numerous bundles of kinocilia (arrowheads). The hair cells are partially ejected from the sensory epithelium (asterisks). The arrow shows an extruded cell body of a hair cell. **Insert in A**: arrowheads indicate small protusions at the surface of the swollen apical pole of a hair cell. **B**: Holes (arrowheads) are seen in the apicalpole of hair cells. The hair cells are partially extruded (asterisk). The arrow shows an extruded hair cell cellular body. **C**: A hair cell partially protrudes in the statocyst cavity (asterisk). **D**: Among some partially extruded hair cells (asterisks), the arrow points to the place left by a totally extruded hair cell. **E**: Upper view of the *msp* of *I. coindetii* shows some holes on the epithelium surface (squares). Cytoplasmic material is extruding (asterisk). Note the center of the macula is free of hair cells in contrast to the macula from other species of cephalopods studied. **Inserts** show some details from E. Arrowhweads indicate disorganized kinocilia. Asteriks show extruding material 2 hair cells that present rupture of the plasma membrane (arrows). **F**: In this area of the *msp*, the cell body of some hair cells is protruding into the statocyst cavity (asterisk) and shows bending kinocillia (arrows). Some hair cells have totally or partially lost their kinocilia (arrowheads). Some holes (squares) are visible on the epithelium. The insert shows a severely damaged area with large holes (squares). **G**: View of the arrangements of the kiociliary groups of the hair cells in regular lines following the epithelium shape on a *macula* of *L. vulgaris*. Because of the uniform orientation per cell, each hair cell is morphologically polarized in just one direction. Note the high density of microvilli. Kinocilia and microvilli form elongated groups. Each kinociliary group represents a single hair cell. Arrowheads show links between the kinocillia that allow polarized movement of the hair cell. **Scale bars: A, F**, G = 10 µm. **B**, **C**, **D**, **inserts in E, inert in** F = 5 µm. E = 50 µm.

Just after sound exposure (Figure 3 A-D) in comparison with the same tissues from control animals (Figure 3G), damage was observed on the *macula statica princeps* (*msp*) sensory epithelium, by SEM analysis. The hair cells were partially ejected from the sensory epithelium. There were spherical holes on the base of the hair cells and a rupture of the plasma membrane, probably due to the extrusion of the internal cellular material. Some hair cells had lost a number of kinocilia or showed bent and flaccid kinocilia.

On animals sacrificed 48h after sound exposure (Figure 3 E-F), the sensory epithelium of the msp presented hair cells partially or totally ejected from the sensory epithelium. The apical ciliated apex and part of the cellular body were extruded above the sensory epithelium into the statocyst cavity. Some hair cells had totally, or in a considerable number, lost the kinocilia and remains of their roots were visible within the damaged epithelium or exhibited bent kinocilia. Large extensions of msp epithelium presented rupture of the plasma membrane on the base of the kinocillia probably due to the swelling and extrusion of the cellular body. The spherical holes observed in animals sacrificed right after the exposure were more pronounced here, confirming the extrusion of the internal cellular material. 

Images of *I. coindetii macula* are shown here. *I. coindetii* presented the center of the msp free of hair cells (Figure 3 E-F). 

### *Loligo vulgaris* and *Illex coindetii crista-cupula* system

**Figure 4:**
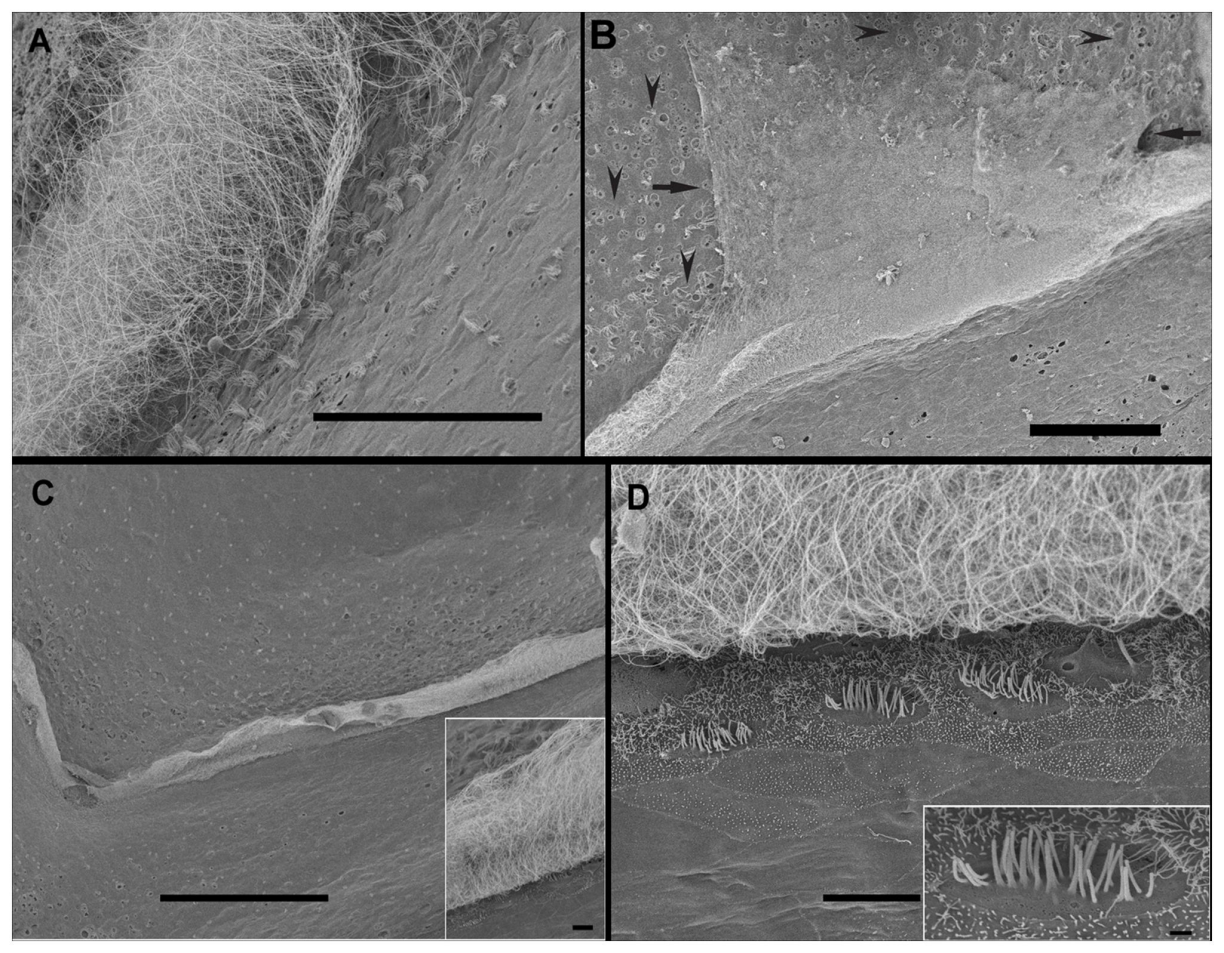
SEM. ***Loligovulgaris* (A, B) and *Illex coindetii* (C, D) cupula. A**, **C**, **D**: **Control animals. B**: **48h after sound exposure. A**: Fibrous *cupula* attached to the *crista*. **B**: The cupula partially adheres to the inner surface of the statocyst (between arrows). Arrowheads show numerous holes on the inner statocyst epithelium near the crista-cupula section. **C**: Upper view of two *I. coindetii* *cupula*. **Insert in C**: Detail of fibrous *cupula* of *I. coindetii* attached to the *crista*. **D**: The contacts of the kinocilia with the fibrous *cupula* are visible. Model of the *I. coindetii* statocyst liner epithelium is shown next to the hair cell rows that surround the main rows of *crista*. *Microvilli* grow in some of the liner epithelium cells. **Insert in D**: Detail of the kinociliary group of a hair cells that surround the main rows of the *crista*. The ground microvilli completely surround the hair cells. **Scale bars**: **A, B, C** = 100 µm. D = 10 µm. **Insert in** B = 5 µm. **Insert in** C = 10 µm. **Insert in** D = 1 µm.

**Figure 5:**
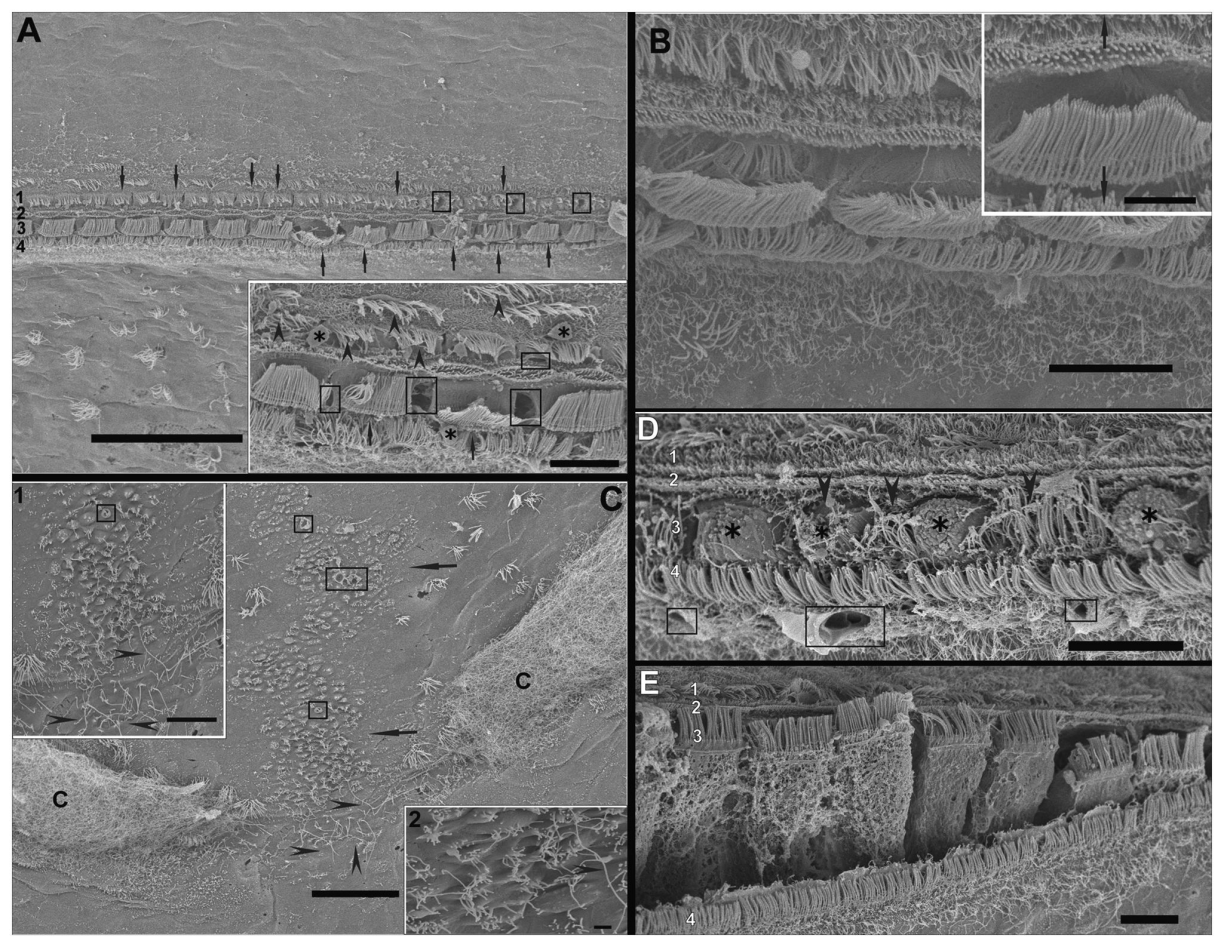
SEM. ***Loligovulgaris* crista sacrificed immediately (A) and *Illexcoindetiicrista* sacrificed 48h (E) after sound exposure**. B: *L.vulgaris crista* (**control animal**). A: amongst the four rows of primary hair cells, two rows (1 and 3) show obvious signs of damage including bending kinocilia (arrows), while row 2 and 4 seem to be more preserved. Some spherical holes (squares) are visible between hair cells of row 1. **Insert**: Detail from A. Arrowheads signs the bending kinocillia of row 1. Squares show the holes on row 3. Note the cellular material extruding (asterisk). *B*: *Crista of L. vulgaris. The four rows of hair cells are visible. The cupula has been partially removed and the kinocilia of the hair cells is now visible. **Insert in B**: Detail of one cell of the two central rows of the crista. Arrows* *indicate* *the* *hair* *cells*’ *direction* *of* *polarization* **C**: A large area of the inner statocyst epithelium between two crista-cupula segments presents cillia (arrows). The cillia are fused in giant cilia (arrowheads). Squares show some spherical holes. Inserts 1, 2 show giant cillia (arrowheads). D: In the acoustically damaged epithelium, the arrangement of hair cells and supporting cells is destructurated. The more severe alterations are seen in hair cells of row 3 and 4. In row 3, the extrusion process of hair cells has started (asterisks). The cellular bodies of hair cells are detached from the epithelium and the apical poles present ruptures of the plasmatic membrane and missing kinocillia. Hair cells show loss of kinocilia or bending or fused kinocillia (arrowheads). Row 4 shows some spherical holes on the base of the hair cells (squares) **E**: In other region, the epithelium is fractured between row 3 and row 4 of hair cells. Note that hair cells in row 3 are partially extruded into the statocyst cavity independently of the neigborouging cells. By contrast, kinocilia on hair cell of row 1, 2 and 4 show a healthy aspect. Scale bars: A = 50 µm. C = 20 µm. B, D, E, **Insert** in A, **Insert in C** (1) = 10 µm. Insert in **C** (2) = 1 µm.

Figures 4 and 5 shows the *crista*-*cupula* system of *I. coindetii*. The *cupula* of *I. coindetii* (Figures 4A, C, D) attached to the *crista* presents a filamentous structure similar to the other decapods. Model of the *I. coindetii* statocyst liner epithelium is shown next to the hair cell rows that surround the main rows of *crista*. Microvilli grow in some of the liner epithelium cells (Figure 4D).

From right after (Figure 5A) until 48h (Figure 5C-E) after sound exposure, in comparison with the same tissues from control animals (Figure 5B), damage was recognized on the *crista* sensory epithelium. Spherical holes could be seen at the base of the hair cells arranged in rows, as well as rupture of the plasma membrane, due to the extrusion of the internal cellular material. As a consequence, apical ciliated hair cells were partially ejected from the sensory epithelium. The damage on kinocilia was not extensive to all the individuals but some individuals showed bent and flaccid kinocilia in their hair cell rows. 

One individual of *I. coindetii* presented the cupula partially adhered to the inner surface of the statocyst (Figure 4B), in comparison with the same tissue from control animals (Figure 4A, C, D). In the same animal, giant cilia (typical on terrestrial animals exposed to sound) are formed by the fusion of the anchored cilia in an area of the inner statocyst epithelium located between two crista-cupula segments (Figure 5C, Inserts in C).

### Lining epithelium of *Illex coindetii* statocyst

**Figure 6:**
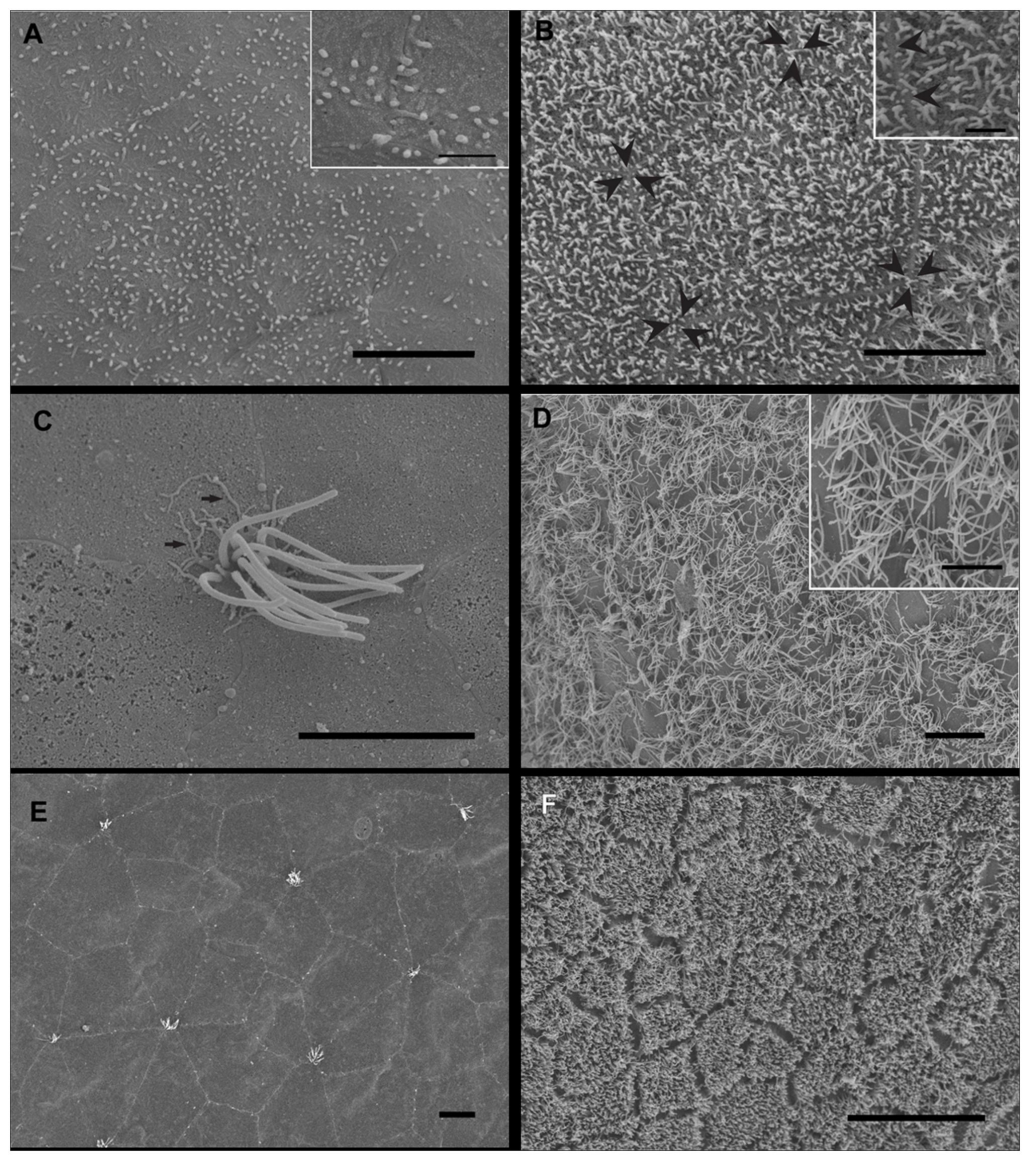
SEM. ***Loligovulgaris* (A-D) and *Illex coindetii* (E, F) inner statocyst structure. Control animals**. The inner surface of decapods statocyst is lined by an epithelium that shows different models. **A**: The flat hexagonal cells of the lining membrane are visible. **Insert in A**: Detail from A. The growing microvilli are visible. **B**: In another area the microvilli are present at high density and cover all the flat hexagonal cells. In A and B it is possible to see the cellular limit and the growing microvilli (arrowheads signs the vertex of the flat hexagonal cells). **Insert in B**: Detail from B. Arrowheads sign the limit of one flat hexagonal cell. **C** shows an individual bundle of cilia (note the root-like structures at the base -arrows-). **D**: A very high density long cilia area is shown. **Insert in D**: Detail from D. The cilia are clearly visible. **E**: In *I. coindetii* the flat hexagonal cells carry cilia on the outer side, which project into the cavity. Microvilli are present in less density than in *L. vulgaris* and surround the epithelium cells. **F**: Another inner statocyst area near the *macula* from *I. coindetii* shows very high density of cilia covering the whole surface of the flat hexagonal cells of the lining epithelium. **Scale bars**: **A, B, C** = 5 µm. **D, E**, F = 10 µm. **Insert in A**, B = 1 µm. **Insert in** D = 5 µm.

Figure 6 shows the lining epithelium of *I. coindetii*. Its features are similar to the other Decapod species inner statocyst structure: flat hexagonal cells carry cilia on the outer side, which project into the cavity. Microvilli are present with less density than in *L. vulgaris* and surround the epithelium cells (Figure 6 E). Other inner statocyst areas near of the *macula* from *I. coindetii* show very high density of cilia covering all the surface of the flat hexagonal cells of the lining epithelium (Figure 6 F).

**Figure 7:**
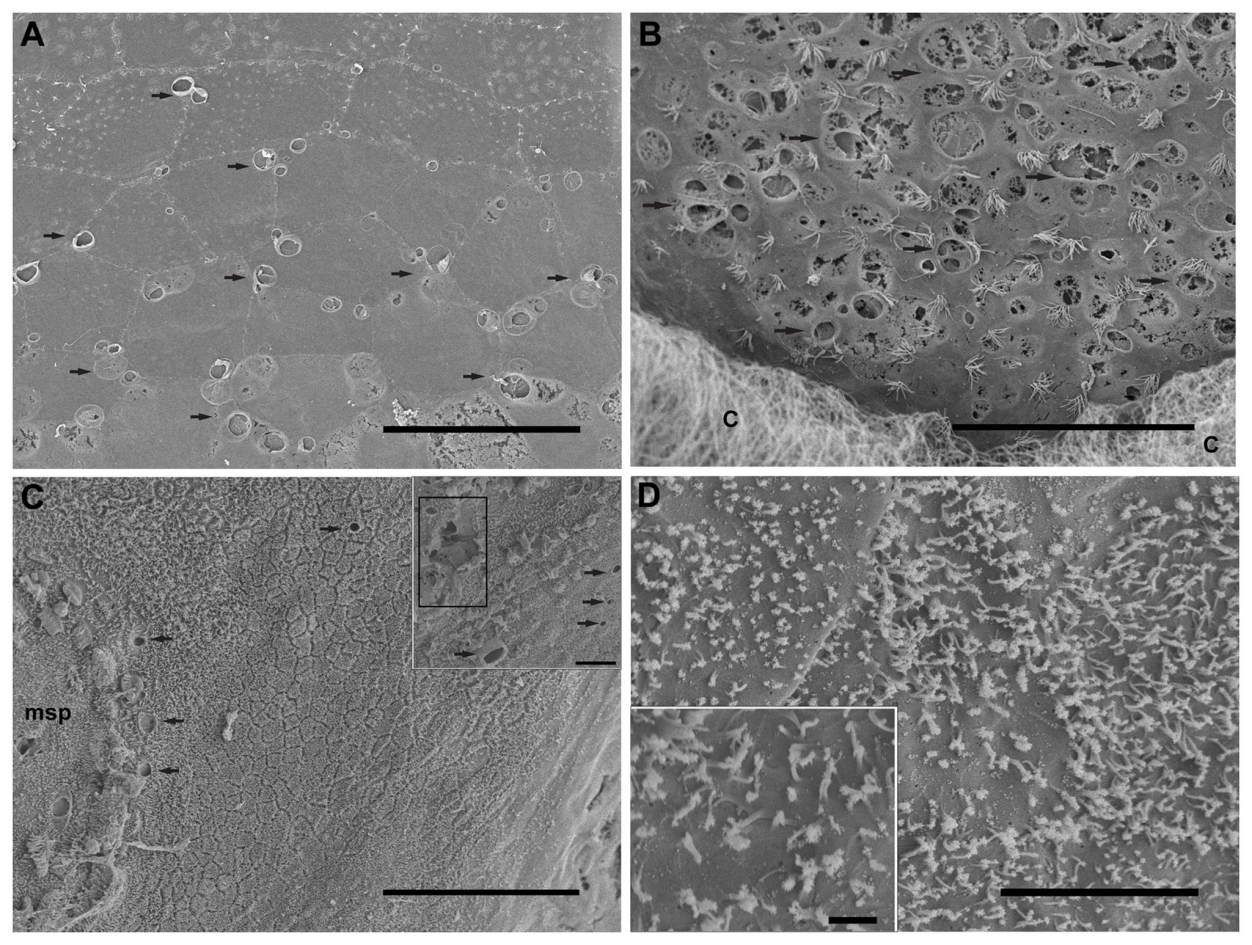
SEM. ***Illex coindetii* lining epithelium of the statocyst cavity, 48h after sound exposure. A**: The cilia are missing and some cells exhibit holes (arrows). **B**: Note the holes (arrowheads) in the epithelial cells and the bending cilia (arrows) on an area near the crista-cupula system (c). **C**: Near the *macula* *statica* *princeps* (msp) a large area shows very high density of cilia covering the whole preserved surface of the flat hexagonal cells of the lining epithelium. In some parts spherical holes (arrows) are visible. **Insert**: arrows show the spherical holes on the epithelium. Some zones are highly damaged (squares). **D**: damaged microvilli form a perimeter surrounding the hexagonal cells. **Insert**: Detail from D. Bending, flaccid and expoiled microvilli. **Scale bars**: **A, B, C**= 50 µm. D = 5 µm. **Insert in** C= 10 µm. **Inserts in** D = 1 µm.

Apart from the specific sensory areas (*msp* and *crista*), in comparison with the same tissues from control animals (Figure 6), the individuals showed acoustic trauma, affecting a wide range of statocyst inner ciliated areas (Figure 7), even on individuals immediately sacrificed after exposure (not shown) but particularly on the individuals sacrificed 48h after exposure (Figure 7). All exposed groups showed lesions in some areas of the lining epithelium of the cavity which consists of flat hexagonal cells with oval nuclei. In some areas of the statocyst, bundles of cilia emerge between the epithelial cells (Figure 6). The lesions in this epithelium consisted basically in the extrusion of the cellular material into the statocyst cavity. In individuals sacrificed at 48 hours after sound exposure there was a massive extension of holes following the extrusion of inner cellular material. The cilia and microvilli were bent flaccid and disorganized in almost all the samples (Figure 7 A-C). In individuals sacrificed 48h some individuals presented some bent, flaccid and expoiled microvilli (Figure 7D).

## Discussion

### Images of *Illex coindetii*

This study shows the first published images of the *macula*, *crista*-*cupula* system and inner statocyst cavity of *I. coindetii*. No previous studies have been carried out on this species. Further analysis of different live stages of *Illex* are needed for a better description of these ultrastructures. As in other decapods, in some parts of its inner cavity the flat hexagonal cells carry cilia on the outer side, which project into the cavity, microvilli are present with less density than in *L. vulgaris* and surround the epithelium cells. The inner statocyst area near the *macula* shows very high density of microvilli covering the whole surface of the flat hexagonal cells of the lining epithelium. The *cupula* of *I. coindetii* attached to the *crista* presents a filamentous structure similar to the other decapods. Model of the *I. coindetii* statocyst liner epithelium is shown next to the hair cell rows that surround the main rows of *crista*. Microvilli grow in some of the liner epithelium cells.

The observation of the statocyst ultrastructure of *Illex* showed a non-previously described feature in cephalopods: the center of the macula princeps presented no hair cells. In other species, it was hypothesized that the macula grows by adding rings of sensory cells from the center to the periphery of the sensory epithelium [36]. Here, *I. coindetii* presents the ciliary groups of the hair cells of the *macula statica princeps* (*msp*) arranged in regular lines, which follow the shape of the epithelium. The microvilli surround the base of the kinocilia. Cilia and microvilli form elongated groups. Each group represents a single hair cell. Every hair cell is arranged in line with an adjacent hair cell only at the macula periphery (Figure 3 E-F). 

The present results suggest there are no hair cells in the center of the macula during the whole *Illex* life. Alternatively, or additionally, rings of hair cells would grow from the periphery to the center of the macula in this species,

### Acoustic Impact

In terrestrial vertebrates (including humans) exposure to very high sound pressure levels may result in permanent hearing loss because the sound destroys sensory hair cells of the inner ear and fractures the bones of the middle ear, in case of blast overpressure [37,38]. Exposure to lower levels for longer periods can also lead to permanent hearing loss due to the death of sensory cells [39]. 

Data on the effects of exposure to sound on fishes is very limited compared with data for terrestrial vertebrates. Some research reported that sound can damage sensory cells in ears of some fish species [40–43]. However, no study has yet determined the relationship between the damage of hair cells and permanent hearing loss in fishes. On the contrary, a post-embryonic recovery of hair cells after noise exposure in some species showed that fish hair cells do regenerate [44,45].

The work of Enger conducted by SEM [40] found that some sensory cells lost their ciliary bundles in the ears of cod (*Gadus morhua*) after 1-5h exposure to pure tones (100-110 dB above threshold in its most sensitive hearing frequency range. Hastings [41,42] reported damage to auditory hair cells in hearing (*Carasius auratus*) happened after exposure to continuous tones (120-140 dB above threshold in its most sensitive hearing frequency range) for approximately 2h, and in oscar (*Astronotus ocellatus*) after 1h of continuous exposure to a 300 Hz pure tone. In this last case the damage was only visible in animals that were alive four days after sound exposure, which allows the conclusion to be drawn that damage caused from exposure to sound takes some time to become visually apparent. Using electron microscopy, McCauley [43] showed destruction of hair cells in ears of pink snapper (*Pagrus auratus*), a sedentary species, after exposure to sound of a seismic air gun. The damage observed in these four species was only a visual manifestation of what may have been a much greater effect. Temporary deafness could result in a fish being unable to respond to presence of predators and to locate preys and mates. It is relevant to mention that several studies showed no damage after exposure to very intense sounds produced by seismic movements [46], and sonar exercices [47]. It was shown that damage to sensory hair cells in fish after very loud pile driving only occurs at sound levels much louder than those that cause other damage to the fish [48].

Because of the very scarce data available in the literature, it is necessary to be extremely cautious when extrapolating results between fish species or received signals, because of the differences in hair cells and hearing systems, the limited data of precise stimulus (pressure and/or particle velocity) and the time course and frequency components of the signals. 

The same considerations may be applied to the studies on the effects of sound on sensory epithelia of cephalopod statocyst. No reference data was available before the current study. This work presents the same morphological and ultrastructural evidence of a massive acoustic trauma induced on individuals belonging to other cephalopod species (*S. officinalis* and *O. vulgaris*) by low frequency sound CEE. The consequences of such CEE are permanent and substantial alterations of the sensory hair cells of the statocysts, the structures responsible for the animals’ sense of balance and position [20,21].

Interestingly, *Illex*, being an epi-mesopelagic species appeared to be affected at a same level. This would mean that the response to low frequency noise would equally alter sensory organs of any species of cephalopods, no matter their foraging ecology. However, because the experimental conditions placed the animals very close to the surface and no variation in pressure levels was performed, the question remains whether this species would also be equally affected when exposed to noise at greater depths.

The lack of any lesion in control animals, or in other organs than the statocyts in exposed individuals, together with the similarity of the injuries found in cuttlefish and octopi after exposure to the same acoustic stimulus, allow us to conclude on a common cause-to-effect relationship between sound and trauma in all exposed individuals. Nevertheless, the acoustic pressure (received) levels reported in this paper cannot be taken as reference values triggering the described lesions, since the laboratory experimental protocol did not include particle motion measurements, nor a precise acoustic mapping of the experimental tank. Future research must address these issues to better understand and define the physics behind the onset of acoustic trauma when cephalopods are exposed to noise.
